# Supplementary material for: Diagnostic Accuracy of Neutrophil Gelatinase-Associated Lipocalin in Peritoneal Effluent and Ascitic Fluid for Early Detection of Peritonitis: A Systematic Review and Meta-Analysis
Source: Med Sci (Basel). 2025 Sep 4;13(3):175. doi: 10.3390/medsci13030175 (PMC12452612; doi:10.3390/medsci13030175)
Supplement: Supplementary file 1 [file medsci-13-00175-s001.zip › medsci-3842728-supplementary.pdf]

Supplementary material

Table S1. PRISMA 2020 checklist for systematic reviews and meta-analyses: items and manuscript locations.
Table S2. Comprehensive descriptive characteristics of the included studies.
Table S3. Comprehensive per-study QUADAS-2 risk-of-bias judgments by domain (Low/Moderate/High) with rationale
Figure S1. **(a)** Study-level sensitivity vs specificity with linear fit and Pearson correlation. **(b)** Deeks' funnel plots under an alternative coding scheme (Haldane–Anscombe +0.5 per cell; ESS defined as $4/(1/TP+1/FN+1/FP+1/TN)$; weighted least-squares regression of ln(DOR) on $1/\sqrt{ESS}$
Figure S2. Subgroup analysis of NGAL diagnostic performance by NGAL test type: **(a)** Forest plot of sensitivity by NGAL test type; **(b)** Forest plot of specificity by NGAL test type: **(c)** Forest plot of diagnostic odds ratio (DOR) by NGAL test type; **(d)** Summary receiver operating characteristic (SROC) curves by NGAL test type.
Figure S3. Leave-one-out influence analysis of the diagnostic odds ratio across NGAL assay platforms and testing contexts: **(a)** ELISA — leave-one-out DOR; **(b)** Point-of-care/rapid tests — leave-one-out DOR; **(c)** Automated immunoassays — leave-one-out DOR; **(d)** Laboratory-based assays (ELISA + automated), collapsed vs POC — leave-one-out DOR; **(e)** POC/rapid tests, collapsed vs laboratory — leave-one-out DOR.
Figure S4. Leave-one-out influence analysis of the diagnostic odds ratio across NGAL assay studies by sample size: **(a)** Studies with less than 100 patients— leave-one-out DOR; **(b)** Studies with more than 100 patients— leave-one-out DOR.

Table S1. PRISMA 2020 Checklist for Systematic Reviews and Meta-Analyses: Items and Manuscript Locations

Section and Topic	Item #	Checklist item	Location where item is reported
**TITLE**			
Title	1	Identify the report as a systematic review.	Title page – first line (identifies as "A Systematic Review and Meta-analysis")
**ABSTRACT**			
Abstract	2	See the PRISMA 2020 for Abstracts checklist.	Structured Abstract (Background, Methods, Results, Conclusions) – page 1
**INTRODUCTION**			
Rationale	3	Describe the rationale for the review in the context of existing knowledge.	Introduction – paragraphs 1–2
Objectives	4	Provide an explicit statement of the objective(s) or question(s) the review addresses.	Introduction – final paragraph (study objective)
**METHODS**			
Eligibility criteria	5	Specify the inclusion and exclusion criteria for the review and how studies were grouped for the syntheses.	§ 2.1 Eligibility Criteria
Information sources	6	Specify all databases, registers, websites, organisations, reference lists and other sources searched or consulted to identify studies. Specify the date when each source was last searched or consulted.	§ 2.2 Search Strategy – first paragraph
Search strategy	7	Present the full search strategies for all databases, registers and websites, including any filters and limits used.	§ 2.2 Search Strategy + Table 1 (full search strings)
Selection process	8	Specify the methods used to decide whether a study met the inclusion criteria of the review, including how many reviewers screened each record and each report retrieved, whether they worked independently, and if applicable, details of automation tools used in the process.	§ 2.3 Study Selection
Data collection process	9	Specify the methods used to collect data from reports, including how many reviewers collected data from each report, whether they worked independently, any processes for obtaining or confirming data from study investigators, and if applicable, details of automation tools used in the process.	§ 2.4 Data extraction and quality assessment of studies
Data items	10a	List and define all outcomes for which data were sought. Specify whether all results that were compatible with each outcome domain in each study were sought (e.g. for all measures, time points, analyses), and if not, the methods used to decide which results to collect.	§ 2.5 paragraphs 1
	10b	List and define all other variables for which data were sought (e.g. participant and intervention characteristics, funding sources). Describe any assumptions made about any missing or unclear information.	§ 2.5 paragraphs 1
Study risk of bias assessment	11	Specify the methods used to assess risk of bias in the included studies, including details of the tool(s) used, how many reviewers assessed each study and whether they worked independently, and if applicable, details of automation tools used in the process.	§ 2.5 paragraphs 6,7,8 (QUADAS-2)<br>3.7 Risk of bias. QUADAS-2, a Deeks' funnel plot, Egger's regression test, funnel plot for diagnostic odds ratio.
Effect measures	12	Specify for each outcome the effect measure(s) (e.g. risk ratio, mean difference) used in the synthesis or presentation of results.	§ 2.5 Statistical Analysis – paragraphs 2-5
Synthesis methods	13a	Describe the processes used to decide which studies were eligible for each synthesis (e.g. tabulating the study intervention characteristics and comparing against the planned groups for each synthesis (item #5)).	§ 3.1 Study Selection– paragraphs 1
	13b	Describe any methods required to prepare the data for presentation or synthesis, such as handling of missing summary statistics, or data conversions.	§§ 2.5 Statistical Analysis - paragraphs 1, 3
	13c	Describe any methods used to tabulate or visually display results of individual studies and syntheses.	§§ 2.6 Data Visualization and Tabulation
	13d	Describe any methods used to synthesize results and provide a rationale for the choice(s). If meta-analysis was performed, describe the model(s), method(s) to identify the presence and extent of statistical heterogeneity, and software package(s) used.	§ 2.5 – Statistical Analysis<br>§ 2.7 Software and Statistical Packages
	13e	Describe any methods used to explore possible causes of heterogeneity among study results (e.g. subgroup analysis, meta-regression).	§ 2.5 – Statistical Analysis paragraphs 4,5,7
	13f	Describe any sensitivity analyses conducted to assess robustness of the synthesized results.	§ 3.4 Subgroup Analysis by Peritonitis Type<br>§ 3.5 Subgroup Analysis by by NGAL Assay Method<br>§ 3.6 Subgroup Analysis by Sample Size

Section and Topic	Item #	Checklist item	Location where item is reported
			Figure 3. Leave-One-Out Sensitivity Analysis of the Pooled Diagnostic Odds Ratio for NGAL in Peritonitis Diagnosis
Reporting bias assessment	14	Describe any methods used to assess risk of bias due to missing results in a synthesis (arising from reporting biases).	3.7 Risk of bias
Certainty assessment	15	Describe any methods used to assess certainty (or confidence) in the body of evidence for an outcome.	3.8 Certainty of the evidence - GRADE-DTA
**RESULTS**			
Study selection	16a	Describe the results of the search and selection process, from the number of records identified in the search to the number of studies included in the review, ideally using a flow diagram.	§3.1 Study Selection+ Figure 1 PRISMA flow diagram
	16b	Cite studies that might appear to meet the inclusion criteria, but which were excluded, and explain why they were excluded.	§ Figure 1 PRISMA flow diagram
Study characteristics	17	Cite each included study and present its characteristics.	§ 3.2 Characteristics of Included Studies - Table 2
Risk of bias in studies	18	Present assessments of risk of bias for each included study.	§ 3.2 QUADAS Risk of Bias – narrative<br>Figure 4. Assessment of publication bias in NGAL diagnostic studies<br>3.7 Risk of bias
Results of individual studies	19	For all outcomes, present, for each study: (a) summary statistics for each group (where appropriate) and (b) an effect estimate and its precision (e.g. confidence/credible interval), ideally using structured tables or plots.	§ 3.3 Performance of Neutrophil Gelatinase-Associated Lipocalin for detection of SBP and PDAP<br>Figure 2 A-D<br>§ 3.4 Subgroup Analysis by Peritonitis Type<br>Figure 5 A-D<br>§ 3.5 Subgroup Analysis by by NGAL Assay Method<br>Figure 6 A-D<br>§ Figure S2. Subgroup analysis of NGAL diagnostic performance by NGAL test type<br>§ 3.6 Subgroup Analysis by Sample Size<br>Figure 7 A-D
Results of syntheses	20a	For each synthesis, briefly summarise the characteristics and risk of bias among contributing studies.	§ 3.7 Risk of bias
	20b	Present results of all statistical syntheses conducted. If meta-analysis was done, present for each the summary estimate and its precision (e.g. confidence/credible interval) and measures of statistical heterogeneity. If comparing groups, describe the direction of the effect.	§ 3.3 Performance of Neutrophil Gelatinase-Associated Lipocalin for detection of SBP and PDAP<br>Figure 2 A-D<br>§ 3.4 Subgroup Analysis by Peritonitis Type<br>Figure 5 A-D<br>§ 3.5 Subgroup Analysis by by NGAL Assay Method<br>Figure 6 A-D<br>§ Figure S2. Subgroup analysis of NGAL diagnostic performance by NGAL test type<br>§ 3.6 Subgroup Analysis by Sample Size<br>Figure 7 A-D
	20c	Present results of all investigations of possible causes of heterogeneity among study results.	§ 3.4 Subgroup Analysis by Peritonitis Type<br>Figure 5 A-D<br>§ 3.5 Subgroup Analysis by NGAL Assay Method<br>Figure 6 A-D<br>§ Figure S2. Subgroup analysis of NGAL diagnostic performance by NGAL test type<br>§ 3.6 Subgroup Analysis by Sample Size<br>Figure 7 A-D

Section and Topic	Item #	Checklist item	Location where item is reported
	20d	Present results of all sensitivity analyses conducted to assess the robustness of the synthesized results.	§ Figure 3. Leave-One-Out sensitivity analysis of the pooled diagnostic odds ratio for NGAL in peritonitis diagnosis
Reporting biases	21	Present assessments of risk of bias due to missing results (arising from reporting biases) for each synthesis assessed.	§ Figure 4. Assessment of publication bias in NGAL diagnostic studies: (a) Deeks' funnel plot asymmetry test; (b) Traditional funnel plot for diagnostic odds ratio.<br>§ 3.4 – 3.7 Risk of bias (Egger's test, funnel plots)
Certainty of evidence	22	Present assessments of certainty (or confidence) in the body of evidence for each outcome assessed.	3.8 Certainty of the evidence<br>Table 3
**DISCUSSION**			
Discussion	23a	Provide a general interpretation of the results in the context of other evidence.	§ 4 Discussion – paragraph 1
	23b	Discuss any limitations of the evidence included in the review.	§ 4 Discussion – paragraph 6
	23c	Discuss any limitations of the review processes used.	§ 4 Discussion – paragraph 4, 6
	23d	Discuss implications of the results for practice, policy, and future research.	§ 4 Discussion – paragraph 5
**OTHER INFORMATION**			
Registration and protocol	24a	Provide registration information for the review, including register name and registration number, or state that the review was not registered.	Title Page & Abstract (PROSPERO CRD420251105563)
	24b	Indicate where the review protocol can be accessed, or state that a protocol was not prepared.	2. Materials and Methods - paragraph 1
	24c	Describe and explain any amendments to information provided at registration or in the protocol.	N/A
Support	25	Describe sources of financial or non-financial support for the review, and the role of the funders or sponsors in the review.	Declarations – Funding (no specific grant)
Competing interests	26	Declare any competing interests of review authors.	Declarations – Disclosure of Interest
Availability of data, code and other materials	27	Report which of the following are publicly available and where they can be found: template data collection forms; data extracted from included studies; data used for all analyses; analytic code; any other materials used in the review.	Declarations – Data availability statement

Table S2. Comprehensive descriptive characteristics of the included studies.

Study (Author, Year, Location)	Study design	Peritonitis type	Participant characteristics	NGAL measurement method	Diagnostic criteria for peritonitis	Diagnostic performance metrics	QUADAS-2 risk of bias
Ahmed et al., 2023 [30], Egypt	Case-cohort study with prospective follow-up	Spontaneous bacterial peritonitis	- Total number of participants: 80 Chronic liver disease patients with ascites<br>- Mean/median age: SBP group: 60.6 ± 12.5 years; Non-SBP group: 57.8 ± 10.2 years<br>- Gender distribution: SBP group: 55% male, 45% female; Non-SBP group: 60% male, 40% female<br>- Specific inclusion criteria: Patients with chronic liver disease and ascites admitted to Internal Medicine Department<br>- Specific exclusion criteria: Acute renal impairment, renal replacement therapy, secondary peritonitis, intra-abdominal surgery or malignancy	- Type of assay: ELISA (SunRed Biotech)<br>- Platform/format: ELISA (laboratory)<br>- Manufacturer/kit: SunRed Biotech<br>- Analyte/target: Total NGAL<br>- Diagnostic cut-off (standardized): 297.8 ng/mL<br>- Original reporting units: ng/mL (standardized to ng/mL)<br>- Cut-off selection rule: reported	- Neutrophil count threshold: PMN ≥ 250 cells/mm³- Microbiological confirmation method: Positive ascitic fluid culture for single organism-<br>- Clinical criteria: Presence of clinical symptoms and signs<br>- Additional diagnostic parameters: Exclusion of acute renal impairment, renal replacement therapy and secondary peritonitis	- Sensitivity: 95.6%<br>- Specificity: 92.5%<br>- Positive Predictive Value: 95%<br>- Negative Predictive Value: 95%<br>- AUC: 0.845<br>- Accuracy: 95%	Domain 1: Patient Selection: Patients were appropriately selected with defined inclusion/exclusion criteria.<br>Domain 2: Index Test: Serum and ascitic Lipocalin-2 were measured by standardized ELISA, with diagnostic performance evaluated by ROC analysis.<br>Domain 3: Reference Standard: SBP was diagnosed by PMN count and/or positive culture.<br>Domain 4: Flow and Timing: Samples were collected at paracentesis, ensuring consistent timing.<br>Overall risk: Moderate
Biomy et al., 2021 [31], Egypt	Cross-sectional study	Spontaneous bacterial peritonitis	- Total number of participants: 85<br>- Patient population: Cirrhosis patients with ascites<br>- Mean age: SBP group: 61±7.81 years; Non-SBP group: 56.44±6.73 years<br>- Gender distribution: SBP group: Male: 28 (66.67%), Female: 14 (22.2%); Non-SBP group: Male: 28 (65.12%), Female: 15 (34.88%)<br>- Specific inclusion/exclusion criteria: Not reported	- Type of assay: ELISA (Bioassay Science Laboratory E1719Hu)<br>- Platform/format: ELISA (laboratory)<br>- Manufacturer/kit: Bioassay Science Laboratory E1719Hu<br>- Analyte/target: Total NGAL<br>- Diagnostic cut-off (standardized): 100.8 ng/mL<br>- Original reporting units: ng/dL (standardized to ng/mL)	- Neutrophil count threshold: PMN >250 cells/mm³<br>- Microbiological confirmation method: Not specified<br>- Clinical criteria: Abdominal pain, fever, GI bleeding<br>- Additional diagnostic parameters: Ascitic fluid protein, glucose, albumin, SAAG	- Sensitivity: 97.62%<br>- Specificity: 97.67%<br>- Positive predictive value: 97.62%<br>- Negative predictive value: 97.67%<br>- AUC: 0.974	Domain 1: Patient Selection: Cirrhotic ascites patients were included with comprehensive exclusion criteria to reduce bias.<br>Domain 2: Index Test: Ascitic NGAL was determined using standardized ELISA, and ROC analysis was used to assess diagnostic efficiency.<br>Domain 3: Reference Standard: SBP was diagnosed by ascitic fluid PMN count ≥250/mm3.<br>Domain 4: Flow and Timing: Ascitic fluid samples were collected consistently for NGAL measurement in relation to SBP diagnosis.<br>Overall risk: High
Chen et al., 2024 [32], China	Multicenter prospective observational study	Peritoneal dialysis-associated peritonitis	- Total number of participants: 221- PD patients from 4 hospitals<br>- Mean/median age: PDAP group: 57.8±13.9 years; Non-PDAP group: 51.2±12.9 years<br>- Gender distribution: PDAP group: 59.5% male, 40.5% female; Non-PDAP group: 59.8% male, 40.2% female<br>- Specific inclusion criteria: Age >18 years, continuous PD treatment ≥3 months<br>- Specific exclusion criteria: Prior antibiotic use before sampling, unclear sample labeling, contaminated samples, repeat cases, red-colored PD effluent	- Type of assay: Rapid immunochromatographic test<br>- Specific kit or technology used: H-NGAL rapid test kit (Qingdao Hantang Biotechnology Co.)- NGAL measurement units: Qualitative (positive/negative)<br>- Test formats: Cassette, strip, and pen types<br>- Reading time: 10-15 minutes	- ISPD criteria (at least 2 of):1) Clinical features: abdominal pain and/or cloudy PD effluent2) PD effluent WBC count >100/µl or >0.1×10⁹/L (dwell time ≥2h) or PMN >50%3) Positive PD effluent culture	- Sensitivity: 100% (95% CI 91.62%-100%)<br>- Specificity: 99.44% (95% CI 96.90%-99.90%)<br>- Accuracy: 99.55% (95% CI 97.48%-99.92%)<br>- Positive Predictive Value: 97.67% (95% CI 87.94%-99.59%)<br>- Negative Predictive Value: 100% (95% CI 97.89%-100%)<br>- Kappa value: 0.985 (95% CI 0.956-1.000)	Domain 1: Patient Selection: Patients were enrolled from multiple centers with clearly defined inclusion and exclusion criteria, ensuring a relevant sample.<br>Domain 2: Index Test: The H-NGAL rapid test was performed by both professionals (blinded) and patients, using various formats to ensure robust evaluation.<br>Domain 3: Reference Standard: PDAP diagnosis strictly adhered to international guidelines, utilizing a combination of clinical, cellular, and microbiological criteria.

							Domain 4: Flow and Timing: Samples were collected at the time of enrollment, and the rapid test results (10-15 minutes) allowed for timely diagnostic assessment. Overall risk: Moderate
Cullaro et al., 2017 [13], USA	Prospective cohort study	Spontaneous bacterial peritonitis	- Total number of participants: 146 - Hospitalized patients with cirrhosis and ascites - Mean/median age: SBP group: 56.6±9.62 years; Non-SBP group: 59.9±10.9 years - Gender distribution: SBP group: 55% male, 45% female; Non-SBP group: 56% male, 44% female- Specific inclusion criteria: Adult patients hospitalized with cirrhosis and ascites undergoing clinically indicated paracentesis - Specific exclusion criteria: Ascites due to non-cirrhotic causes, recent abdominal surgery, solid organ transplant recipients, documented colitis or enteritis	- Type of assay: ELISA - Specific kit or technology used: AntibodyShop, Gentofte, Denmark - NGAL measurement units: ng/mL - Limit of detection: 0.5-4.0 ng/mL Diagnostic cut-off (standardized): 230.05 ng/mL - Original reporting units: ng/mL - Intra-assay variation: 2.1% (range 1.3-4.0)	Neutrophil count threshold: ANC ≥250 cells/mm³ - Microbiological confirmation method: Blood, urine, or ascites cultures - Clinical criteria: Not specified- Additional diagnostic parameters: None	- For SBP diagnosis: - c-statistic (AUC): 0.68 - For mortality prediction: Sensitivity: 73.3% (cutoff >221.3 ng/mL), Specificity: 71.2% (cutoff >221.3 ng/mL), AUC: 0.79	Domain 1: Patient Selection: Low risk - consecutive patients with clear inclusion/exclusion criteria Domain 2: Index Test: Low risk - ELISA performed with standardized protocol, blinded to reference standard Domain 3: Reference Standard: Low risk - ANC ≥250 cells/mm³ is standard criterion Domain 4: Flow and Timing: Low risk - samples collected on day of paracentesis Overall risk: Low
Hassan et al., 2023 [19], Egypt	Case-control study	Spontaneous bacterial peritonitis	-Total number of participants: 72 - Patients with cirrhotic liver and ascites- Mean/median age: Not reported - Gender distribution: Not reported- Specific inclusion criteria: All patients diagnosed as cirrhotic based on clinical and laboratory tests with ascites caused by chronic liver illness - Specific exclusion criteria: Cirrhotic patients with HCC, peritonitis due to any cause other than SBP, portal hypertension and ascites from non-cirrhotic causes, liver or organ transplantation, renal diseases	- Type of assay: ELISA - Specific kit or technology used: Human Lipocalin linked with Neutrophil Gelatinase Kit, Sun Red bio company, China - NGAL measurement units: ng/mL - Threshold values used for diagnosis: ≥230.05 ng/mL	- Neutrophil count threshold: PMNL count ≥250/mm³ - Microbiological confirmation method: Positive fluid cultures with single organism culture isolation - Clinical criteria: Clinical presence of ascites, hepatic encephalopathy, jaundice or hematemesis and melena - Additional diagnostic parameters: Laboratory tests (CBC platelet <150000, liver function test albumin<3.5, INR >1.1)	- Sensitivity: 94.4% - Specificity: 88.9% - Positive Predictive Value: 89.5% - Negative Predictive Value: 94.1% - AUC: 0.989- Accuracy: 91.7%	Domain 1: Patient Selection: Moderate risk - case-control design with clear inclusion/exclusion criteria Domain 2: Index Test: Low risk - ELISA performed with standardized protocol Domain 3: Reference Standard: Low risk - PMNL count ≥250/mm³ and/or positive culture Domain 4: Flow and Timing: Low risk - samples collected at paracentesis Overall risk: Moderate
Khalil et al., 2023 [33], Egypt	Case-control study	Spontaneous bacterial peritonitis and Culture-negative neutrocytic ascites	-Total number of participants: 150 - Cirrhotic patients divided into infected (n=100) and non-infected (n=50) - SBP subgroup: 55 patients- CNNA subgroup: 15 patients- UTI subgroup: 30 patients- Mean/median age: Not reported- Gender distribution: Not reported - Specific inclusion criteria: Patients aged ≥18 years with liver cirrhosis and ascites - Specific exclusion criteria: Antibiotic treated patients, renal failure, HCC, malignant ascites,	- Type of assay: ELISA (DRG GmbH) - Platform/format: ELISA (laboratory) - Manufacturer/kit: DRG GmbH - Analyte/target: Total NGAL - Diagnostic cut-off (standardized): 110.72 ng/mL - Original reporting units: ng/mL (standardized to ng/mL) - Cut-off selection rule: reported	- Neutrophil count threshold: AF neutrophils count ≥250×10³ cells/μL - Microbiological confirmation method: Positive ascitic fluid culture for SBP; negative culture for CNNA - Clinical criteria: Clinical symptoms and signs - Additional diagnostic parameters: Mac-1 expression by flow cytometry	- Sensitivity: 92.7% (CI: [0.82, 0.98]) - Specificity: 84% (CI: [0.75, 0.91]) - Positive Predictive Value: 0.77 (CI: [0.65, 0.86]) - Negative Predictive Value: 0.95 (CI: [0.88, 0.98]) - Area under the ROC curve (AUC): 0.899 (CI: [0.848, 0.951])	Domain 1: Patient Selection: Moderate risk - case-control design with clear inclusion/exclusion criteria Domain 2: Index Test: Low risk - ELISA and flow cytometry performed with standardized protocols Domain 3: Reference Standard: Low risk - standard criteria for SBP and CNNA Domain 4: Flow and Timing: Low risk - samples collected within 6h after paracentesis Overall risk: Moderate

			septicemia, secondary bacterial peritonitis				
Lippi et al., 2013 [20], Italy	Cross-sectional study	Bacterial peritonitis (various causes)	- Total number of participants: 111<br>- Patient population: Patients with new onset nonmalignant ascites<br>- Mean/median age: Not reported<br>- Gender distribution: Not reported<br>- Specific inclusion criteria: Consecutive peritoneal fluids from patients with new onset nonmalignant ascites<br>- Specific exclusion criteria: Visible clots in samples (7 samples excluded)	- Type of assay: Automated NGAL Test™ (BioPorto Diagnostics A/S) on Beckman Coulter AU5822<br>- Platform/format: Immunoturbidimetric (laboratory)<br>- Manufacturer/kit: BioPorto Diagnostics A/S<br>- Analyte/target: Total NGAL<br>- Diagnostic cut-off (standardized): 120.0 ng/mL	- Neutrophil count threshold: PMN ≥250/μL<br>- Microbiological confirmation method: Not used (68% on antibiotics)<br>- Clinical criteria: New onset nonmalignant ascites<br>- Additional diagnostic parameters: LDH, proteins, glucose	- Sensitivity: 96% (95% CI: 80-100%)<br>- Specificity: 75% (95% CI: 65-84%)<br>- Positive Predictive Value: Not reported<br>- Negative Predictive Value: Not reported<br>- AUC: 0.89 (95% CI: 0.82-0.95)<br>- Accuracy: Not reported	Domain 1: Patient Selection: Low risk - consecutive patients with defined criteria<br>Domain 2: Index Test: Low risk - predefined threshold, automated assay<br>Domain 3: Reference Standard: Moderate risk - PMN count used instead of culture due to antibiotic use<br>Domain 4: Flow and Timing: Low risk - all samples analyzed similarly<br>Overall risk: Moderate
Liu et al., 2020 [21], China	Prospective cohort	Spontaneous bacterial peritonitis	-Total number of participants: 204 decompensated liver cirrhosis patients with ascites<br>- Mean/median age: Non-SBP group: 57.31 ± 12.91 years; SBP group: 59.64 ± 11.95 years<br>- Gender distribution: Non-SBP group: 65.7% male, 34.3% female; SBP group: 76.6% male, 23.4% female<br>- Specific inclusion criteria: Consecutive hospitalized patients with decompensated liver cirrhosis and ascites<br>- Specific exclusion criteria: Pre-existing renal disease, presence of AKI at hospitalization, renal replacement therapy, secondary peritonitis, malignant diseases	- Type of assay: Latex-enhanced immunoturbidimetric (BSBE)<br>- Platform/format: Immunoturbidimetric (laboratory)<br>- Manufacturer/kit: BSBE<br>- Analyte/target: Total NGAL<br>- Diagnostic cut-off (standardized): 108.95 ng/mL	- Neutrophil count threshold: PMN ≥ 250 cells/mm^3 and/or positive ascitic fluid culture<br>- Microbiological confirmation method: Ascitic fluid culture (positive in 9 patients: 7 *E. coli, 2 K. pneumoniae*)<br>- Clinical criteria: Clinical and biological diagnosis of decompensated liver cirrhosis	- Sensitivity: 76.9% (for mortality prediction)<br>- Specificity: 45.1% (for mortality prediction)<br>- Positive Predictive Value: Not reported<br>- Negative Predictive Value: Not reported<br>- AUC: 0.702 (for mortality prediction in SBP patients)<br>- Accuracy: Not reported	Domain 1: Patient Selection: Consecutive patients with well-defined inclusion/exclusion criteria.<br>Domain 2: Index Test: Ascitic NGAL measured by standardized latex-enhanced immunoturbidimetric method, with ROC analysis for diagnostic performance.<br>Domain 3: Reference Standard: SBP diagnosed by PMN ≥ 250 cells/mm^3 and/or positive ascitic fluid culture.<br>Domain 4: Flow and Timing: Ascitic samples collected at paracentesis with consistent timing for all patients.<br>Overall risk: Low
Martino F. et al., 2012 [34], Italy	Case–control study	Peritoneal dialysis-associated peritonitis	- Total number of participants: 60 (30 cases, 30 controls)<br>- Patient population: PD patients on treatment >3 months- Mean/median age: Cases: 66.5 years (IQR 52.2-73); Controls: 70 years (IQR 59.7-73.2)<br>- Gender distribution: Cases: 73.3% male; Controls: 73.3% male<br>- Specific inclusion criteria: PD patients >3 months with signs/symptoms of peritonitis (cases) or routine visit (controls)<br>- Specific exclusion criteria: Not reported	Type of assay: Chemiluminescent microparticle immunoassay<br>- Specific kit or technology used: Architect platform (Abbott Diagnostics)<br>- NGAL measurement units: ng/mL<br>- Threshold values used for diagnosis: Not specified (ROC analysis performed)	ISPD guidelines: Cloudy peritoneal effluent with WBC > 100 × 10^6 cells/L (after ≥ 2 h dwell) with > 50% PMNs, abdominal pain and/or positive culture or Gram stain	- Sensitivity: Not reported- Specificity: Not reported- Positive Predictive Value: Not reported- Negative Predictive Value: Not reported<br>-AUC peritoneal NGAL 0.99 ($p < 0.001$);AUC CRP 0.81 ($p = 0.001$), PCT 0.70 ($p = 0.039$), WBC 1.00 ($p < 0.001$)	Domain 1: Patient Selection: Low risk - consecutive cases and matched controls<br>Domain 2: Index Test: Unclear risk - no predefined threshold reported<br>Domain 3: Reference Standard: Low risk - standard ISPD criteria used<br>Domain 4: Flow and Timing: Low risk - appropriate sample collection<br>Overall risk: Moderate
Martino et al., 2015 [22], Italy	Case-control study	Peritoneal dialysis-associated peritonitis	- Total number of participants: 182 (91 cases, 91 controls)<br>- Patient population: PD patients on treatment ≥90 days	- Type of assay: Chemiluminescent microparticle immunoassay<br>- Specific kit or technology	- Neutrophil count threshold: WBC > 100 cells/mm^3 (with at least	- Sensitivity: 90%<br>- Specificity: 82%<br>- Positive predictive value: Not reported	Domain 1: Patient Selection: Low risk - consecutive enrollment with clear criteria

			- Mean/median age: Peritonitis: 65 years (IQR 53.7-73.7); No peritonitis: 66.5 years (IQR 53.25-78)- Gender distribution: Peritonitis: 77.5% male; No peritonitis: 71.6% male- Specific inclusion criteria: Age >18 years, PD treatment ≥90 days, informed consent<br>- Specific exclusion criteria: Peritonitis episode within 30 days before enrollment	used: Architect platform (Abbott Diagnostics)<br>- NGAL measurement units: ng/mL<br>- Threshold values used for diagnosis: 85 ng/mL	50% polymorphonuclear cells)<br><br>- Microbiological confirmation method: Positive culture or Gram stain<br><br>- Clinical criteria: Clinical signs and symptoms of peritoneal inflammation (pain, discomfort, nausea/vomiting, diarrhea, or constipation)<br><br>- Additional diagnostic parameters: Cloudy drainage	- Negative predictive value: Not reported<br>- Area under the ROC curve (AUC): 0.936<br>- Accuracy: Not reported	Domain 2: Index Test: Low risk - ROC-determined threshold, blinded analysis<br>Domain 3: Reference Standard: Low risk - standard ISPD criteria<br>Domain 4: Flow and Timing: Low risk - appropriate sample collection<br>Overall risk: Low.
Morisi et al., 2024 [35], Italy	Retrospective analysis with cross-sectional elements	Peritoneal dialysis-associated peritonitis	- Total number of participants: 301 peritoneal effluent samples<br>- Patient population: PD patients (stable and with suspected/confirmed peritonitis)<br>- Mean/median age: Not reported-Gender distribution: Not reported<br>- Specific inclusion criteria: PD patients undergoing routine analysis or with suspected/confirmed peritonitis<br>- Specific exclusion criteria: Not reported	- Type of assay: Dipstick (NGALds) vs turbidimetric (NGALlab)<br>- Platform/format: POC rapid (dipstick/lateral flow)<br>- Manufacturer/kit: NGALds<br>- Analyte/target: Total NGAL<br>- Diagnostic cut-off (standardized): 100.0 ng/mL<br>- Original reporting units: μg/L (standardized to ng/mL)	- Neutrophil count threshold: White cell count and percentage of neutrophils (specific thresholds not reported)<br>- Microbiological confirmation method: Not specified<br>- Clinical criteria: ISPD criteria- Additional diagnostic parameters: NGALlab used as comparator	- Sensitivity: 96%<br>- Specificity: Not reported<br>- Positive predictive value: 0.64<br>- Negative predictive value: 0.87<br>- Area under the ROC curve (AUC): 0.82	Domain 1: Patient Selection: Unclear risk - retrospective design, selection criteria not fully described<br>Domain 2: Index Test: Low risk - threshold determined by statistical analysis<br>Domain 3: Reference Standard: Unclear risk - reference standard not fully described<br>Domain 4: Flow and Timing: Low risk - parallel testing performed<br>Overall risk: Moderate
Virzì et al., 2022 [36], Vicenza,Italy	Observational, case–control study	Dialysis associated peritonitis	- Total number of participants = 30 PD patients (17 with peritonitis; 13 controls) - Age ≥ 18 years; on PD ≥ 30 days; informed consent - PD modality: 7 CAPD, 23 APD; treatment duration median 32.7 mo (IQR 11–49; range 1–88 mo) - Comorbidities: 9/30 diabetes; 30/30 hypertension; 12/30 CVD; none immunosuppressed; 86.6% on erythropoietin	Laboratory-based NGAL: particle-enhanced turbidimetric immunoassay (BioPorto Diagnostics; range 50–3000 ng/mL; ≥ 200 ng/mL indicative of peritonitis).<br>Point-of-care NGAL (NGALds): lateral-flow dipstick (BioPorto Diagnostics), semi-quantitative colour categories 25–600 ng/mL; read by two blinded operators	ISPD guidelines: Cloudy peritoneal effluent with WBC > 100 × 10^6 cells/L (after ≥ 2 h dwell) with > 50% PMNs, abdominal pain and/or positive culture or Gram stain	Spearman's ϱ between NGALds and lab NGAL = 0.88 ($p < 0.01$), Spearman's ϱ between NGALds and effluent WCC = 0.82 ($p < 0.01$), Inter-operator reproducibility: ϱ = 0.847 ($p < 0.001$), κ = 0.786 ($p < 0.001$). All peritonitis cases had NGALds ≥ 300 ng/mL (proposed cutoff) *No formal sensitivity/specificity or AUC reported (preliminary study)*	Domain 1: Patient selection: clear inclusion/exclusion; consecutive enrolment<br>Domain 2: Index test: blinded operators; pre-specified cutoffs<br>Domain 3: Reference standard: ISPD criteria with culture/WCC<br>-Flow & timing: concurrent sampling for cases/controls<br>Domain 4: Interpretation: objective readouts (dipstick categories; immunoassay thresholds)<br>Overall risk: High
Virzì et al., 2024 [37], Italy	Retrospective analysis	Peritoneal dialysis-associated peritonitis	- Total number of participants: 301 peritoneal effluent samples<br>- Patient population: PD patients (healthy during routine analysis and with suspected/confirmed peritonitis)<br>- Mean/median age: Not reported	Type of assay: Two methods compared: NGALlab: BioPorto test (particle-enhanced turbidimetric immunoassay)	Diagnosed by ISPD criteria for peritonitis	- Spearman's Rs = 0.876 ($p = 10^{-96}$)<br>- Sensitivity: 96 % (150/156)<br>- Specificity, PPV, NPV, AUC: not reported	- Domain 1: Patient Selection - Low risk (parallel samples from both healthy and peritonitis patients)<br>- Domain 2: Index Test - Low risk (NGALds compared to established NGALlab)

			- Gender distribution: Not reported<br>- Specific inclusion criteria: PD patients undergoing routine analysis or with suspected/confirmed peritonitis<br>- Specific exclusion criteria: Not reported	NGALds: Rapid semi-quantitative colorimetric dipstick test<br>- Specific kit or technology used: Antibody sandwich lateral flow dipstick test-NGAL measurement units: µg/L<br>- Threshold values used for diagnosis: >100 µg/L for peritonitis diagnosis-Dipstick categories: 25, 50, 100, 150, 300, 600 µg/L			- Domain 3: Reference Standard - Low risk (NGALlab as reference)<br>- Domain 4: Flow and Timing - Low risk (retrospective analysis with parallel samples)<br>Overall risk: Moderate	

ANC, Absolute Neutrophil Count; APD, Automated Peritoneal Dialysis; AUC, Area Under the Curve; CAPD, Continuous Ambulatory Peritoneal Dialysis; CBC, Complete Blood Count; CI, Confidence Interval; CNNA, Culture-Negative Neutrocytic Ascites; CRP, C-Reactive Protein; CVD, Cardiovascular Disease; ELISA, Enzyme-Linked Immunosorbent Assay; GI, Gastrointestinal; HCC, Hepatocellular Carcinoma; INR, International Normalized Ratio; IRB, Institutional Review Board; ISPD, International Society for Peritoneal Dialysis; LDH, Lactate Dehydrogenase; NGAL, Neutrophil Gelatinase-Associated Lipocalin; NGALds, NGAL Dipstick Test; NGALlab, Laboratory-based NGAL Test; NPV, Negative Predictive Value; PCT, Procalcitonin; PD, Peritoneal Dialysis; PDAP, Peritoneal Dialysis-Associated Peritonitis; PMN, Polymorphonuclear Neutrophils; PMNL, Polymorphonuclear Leukocytes; PPV, Positive Predictive Value; QUADAS-2, Quality Assessment of Diagnostic Accuracy Studies-2; ROC, Receiver Operating Characteristic; SAAG, Serum-Ascites Albumin Gradient; Ascites Serum; SBP, Spontaneous Bacterial Peritonitis; UTI, Urinary Tract Infection; WBC, White Blood Cell; WCC, White Cell Count.

Table S3. Comprehensive per-study QUADAS-2 risk-of-bias judgments by domain (Low / Moderate / High) with rationale

Study	D1 – Patient selection	D2 – Index test	D3 – Reference standard	D4 – Flow & timing	Overall
Ahmed et al., 2023 (Egypt)	Low — consecutive cirrhotic ascites; clear inclusion/exclusion.	Moderate — cut-off derived from ROC (not pre-specified).	Low — SBP per guidelines (PMN ≥250 cells/μL ± culture).	Low — sampling at paracentesis; minimal delay.	Moderate
Biomy et al., 2021 (Egypt)	Moderate — cross-sectional; consecutiveness not explicit.	Moderate — ROC-based threshold (data-driven).	Low — guideline-concordant SBP definition.	Low — index and reference obtained same encounter.	High
Chen et al., 2024 (China)	Low — multicentre cohort with explicit IC/EC.	Low — POC H-NGAL; operator blinding; fixed procedure.	Low — ISPD criteria for PD-peritonitis.	Low — 10–15 min read-out; near-simultaneous RS.	Moderate
Cullaro et al., 2017 (USA)	Low — prospective cohort; consecutive enrolment.	Low — ELISA; interpretation blinded to RS.	Low — PMN ≥250 cells/μL as RS.	Low — ascites tested at paracentesis.	Low
Hassan et al., 2023 (Egypt)	High — case–control design.	Low — ELISA under standard lab procedures.	Low — guideline-concordant RS.	Low — same-visit sampling/assessment.	Moderate
Khalil et al., 2023 (Egypt)	High — case–control (infected vs non-infected).	Low — standardized ELISA/flow methods.	Low — SBP/CNNA per standard definitions.	Low — adequate specimen timing/handling.	Moderate
Lippi et al., 2013 (Italy)	Moderate — new-onset ascites; partial spectrum concerns.	Low — automated assay; fixed analytical protocol.	Low — PMN-based RS (culture often limited by prior antibiotics).	Low — single-visit assessment.	Moderate
Liu et al., 2020 (China)	Low — prospective cirrhosis cohort.	Moderate — latex-turbidimetry; ROC-derived cut-off.	Low — PMN ≥250 cells/μL ± culture.	Low — same-day testing.	Low
Martino F. et al., 2012 (Italy)	Moderate — small PD cohort; sampling frame not explicit.	Moderate — post-hoc (ROC) threshold.	Low — ISPD peritonitis criteria.	Low — specimen and RS close in time.	Moderate
Martino et al., 2015 (Italy)	High — case–control PD peritonitis vs controls.	Low — assay procedures described; blinding stated.	Low — ISPD/WBC reference standard.	Low — same-session collection.	Low
Morisi et al., 2024 (Italy)	Moderate — retrospective PD samples; convenience set.	Low — dipstick vs lab NGAL; predefined positivity rule.	Low — ISPD/WBC as RS.	Low — parallel measurements.	Moderate
Virzì et al., 2022 (Vicenza, Italy)	Moderate — feasibility/validation with convenience sampling.	Low — NGAL-dipstick vs lab; predefined readout.	Low — ISPD/WBC reference.	Low — simultaneous/near-simultaneous measures.	High
Virzì et al., 2024 (Italy)	Moderate — PD validation; partial sampling frame.	Low — dipstick vs lab; fixed protocol.	Low — ISPD/WBC reference.	Low — paired measurements.	Moderate

Figure S1. **(a)** Study-level sensitivity vs specificity with linear fit and Pearson correlation. **(b)** Deeks' funnel plots under an alternative coding scheme (Haldane–Anscombe +0.5 per cell; ESS defined as $4/(1/TP+1/FN+1/FP+1/TN)$; weighted least-squares regression of ln(DOR) on $1/\sqrt{ESS}$

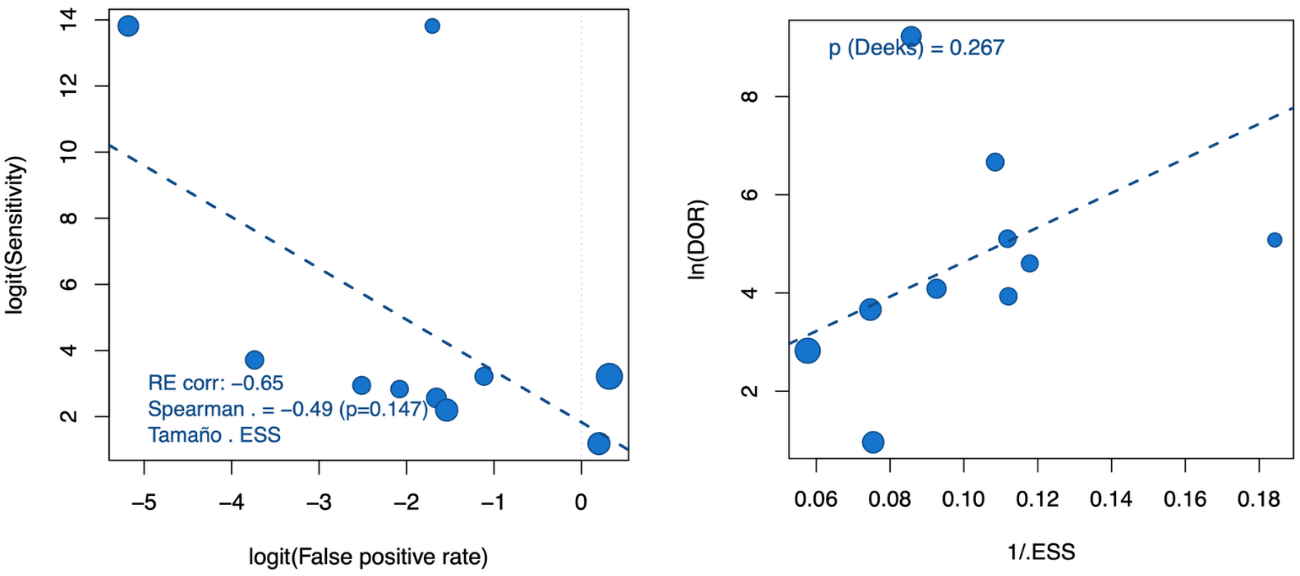

(**a**) (**b**)

The slope test for the overall dataset was not significant (p = 0.27), indicating that evidence for small-study effects is sensitive to analytic choices given the small number of studies.

Figure S2. Subgroup analysis of NGAL diagnostic performance by NGAL test type: **(a)** Forest plot of sensitivity by NGAL test type; **(b)** Forest plot of specificity by NGAL test type: **(c)** Forest plot of diagnostic odds ratio (DOR) by NGAL test type; **(d)** Summary receiver operating characteristic (SROC) curves by NGAL test type.

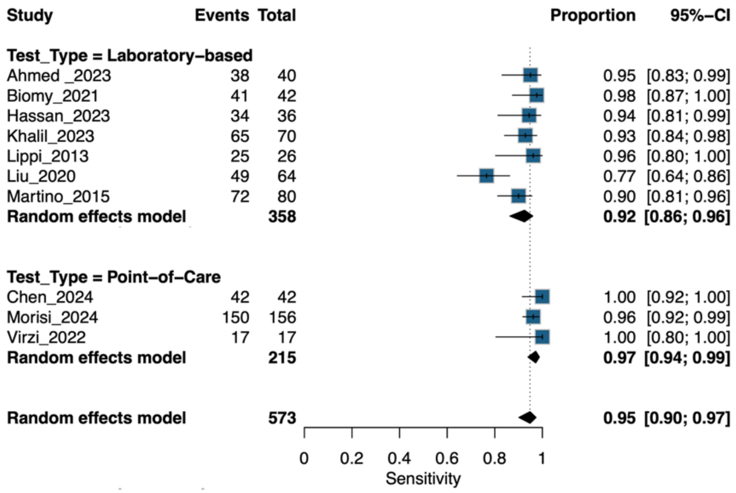

(**a**)

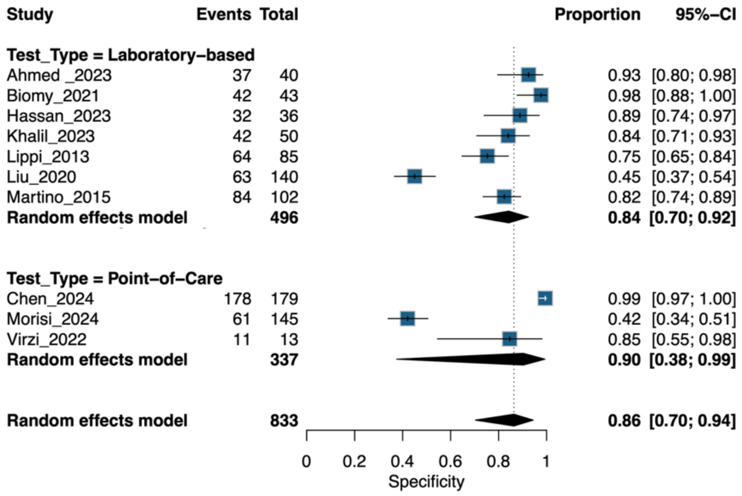

(**b**)

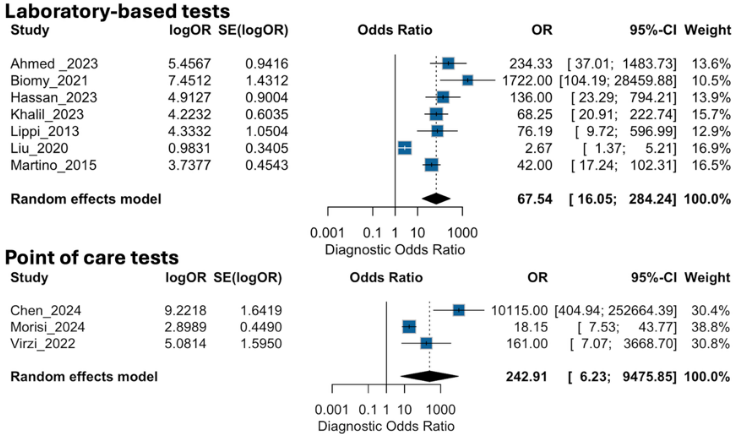

(**c**)

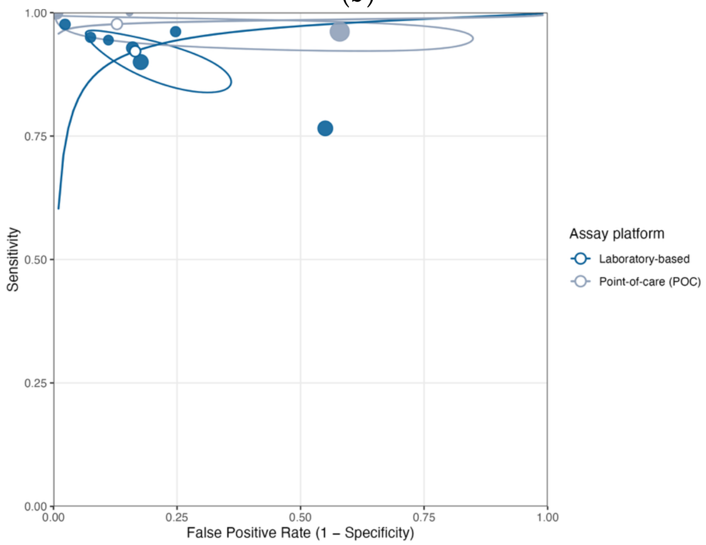

(**d**)

Figure S3. Leave-one-out influence analysis of the diagnostic odds ratio across NGAL assay platforms and testing contexts: **(a)** ELISA — leave-one-out DOR; **(b)** Point-of-care/rapid tests — leave-one-out DOR; **(c)** Automated immunoassays — leave-one-out DOR; **(d)** Laboratory-based assays (ELISA + automated), collapsed vs POC — leave-one-out DOR; **(e)** POC/rapid tests, collapsed vs laboratory — leave-one-out DOR.

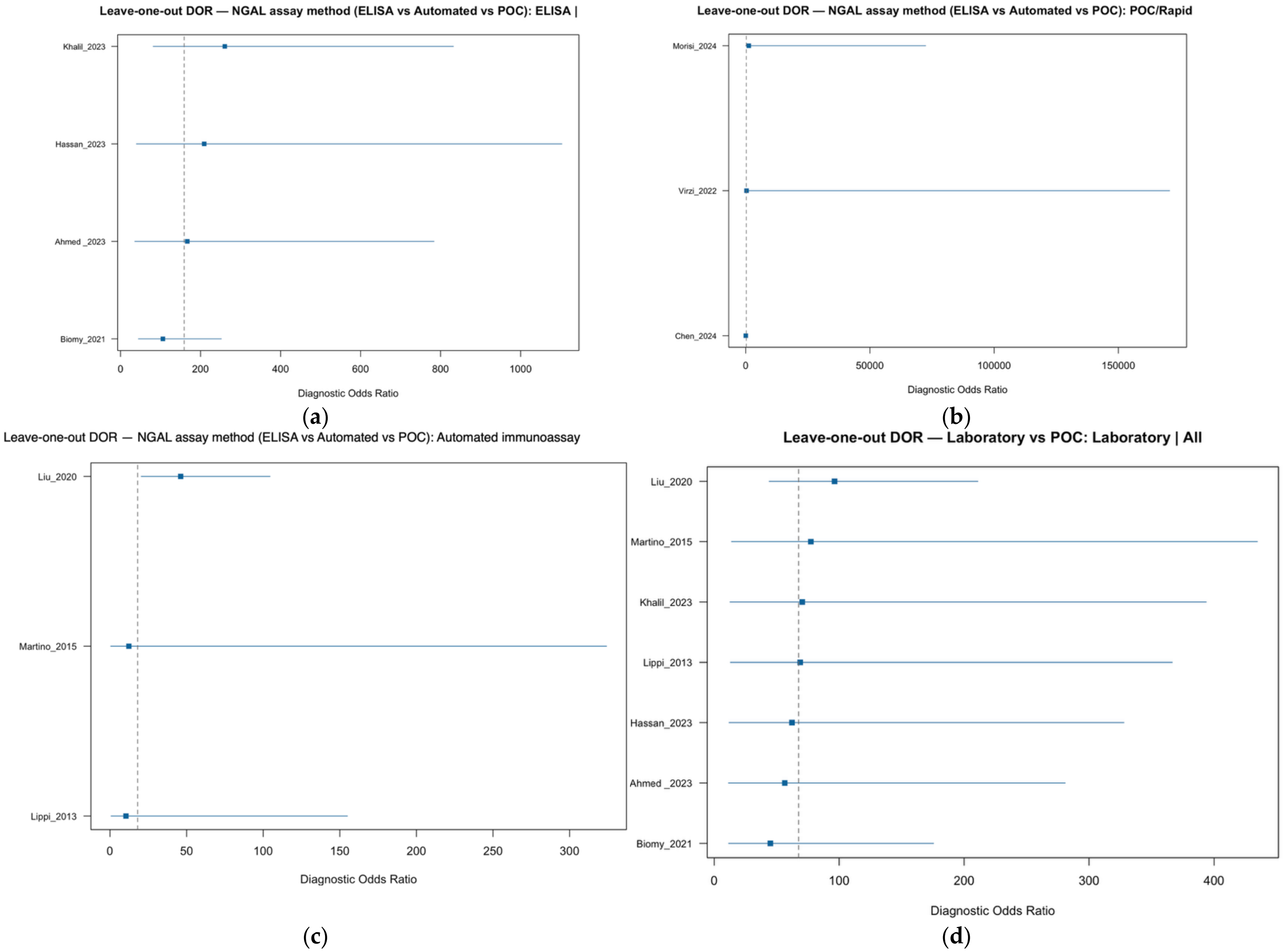

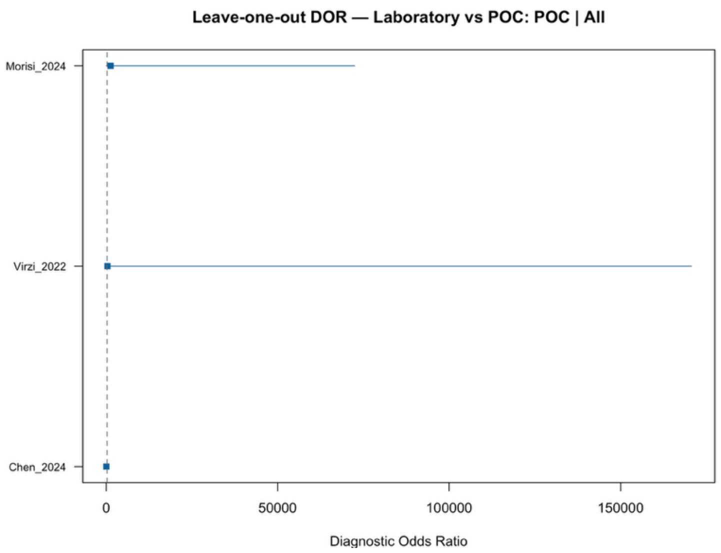

(**e**)

Abbreviations: DOR, diagnostic odds ratio; ELISA, enzyme-linked immunosorbent assay; POC, point-of-care; NGAL, neutrophil gelatinase-associated lipocalin.

Figure S4. Leave-one-out influence analysis of the diagnostic odds ratio across NGAL assay studies by sample size: **(a)** Studies with less than 100 patients—leave-one-out DOR; **(b)** Studies with more than 100 patients— leave-one-out DOR.

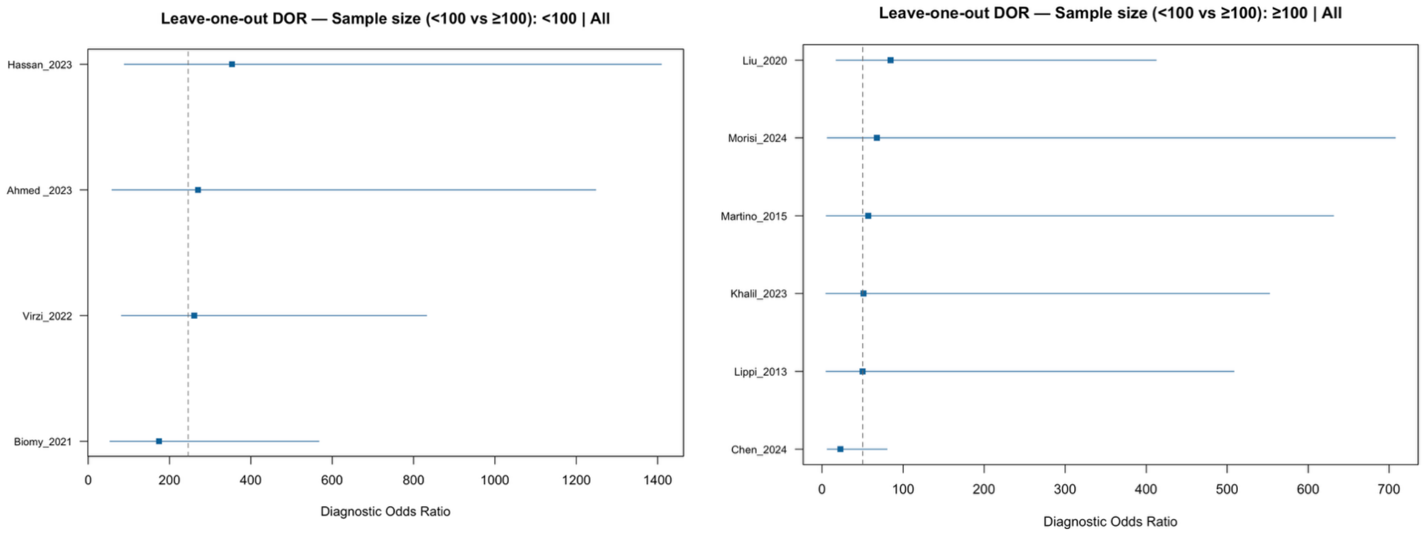

(**a**) (**b**)

Abbreviations: DOR, diagnostic odds ratio
